# Use of spectral indices and photosynthetic parameters to evaluate the growth performance of hydroponic tomato at different salinity levels

**DOI:** 10.1371/journal.pone.0325839

**Published:** 2025-06-06

**Authors:** Khalid A. Al-Gaadi, Ahmed M. Zeyada, Elkamil Tola, Rangaswamy Madugundu, Mohamed K. Edrris, Omer Mahjoop

**Affiliations:** 1 Department of Agricultural Engineering, College of Food and Agriculture Sciences, King Saud University, Riyadh, Saudi Arabia; 2 Precision Agriculture Research Chair, Deanship of Scientific Research, King Saud University, Riyadh, Saudi Arabia; United Arab Emirates University, UNITED ARAB EMIRATES

## Abstract

Conventional methods for measuring plant physiological parameters are expensive and time-consuming, and this has promoted the use of optical and sensing techniques. Therefore, this study was conducted to investigate the effect of salinity on the performance of hydroponic tomato plants, based on optical and sensing techniques (i.e., spectral indices and photosynthetic parameters), as well as fruit yield. Four spectral vegetation indices-VIs (Moisture Stress Index “MSI”, Canopy Response Salinity Index “CRSI”, Normalized Difference Nitrogen Index “NDNI” and Green Leaf Index “GLI”) were calculated using spectral measurements collected from tomato plant leaves. Also, four photosynthetic parameters (Net photosynthetic rate “P_N_”, Water use efficiency “WUE”, Transpiration rate “Tr” and Total stomatal conductance “Gs”) were measured from the same tomato plant leaves. Measurements were recorded for tomato plants grown under three salinity levels (Salinity-1; 2.5 dS m^-1^), (Salinity-2; 4.0 dS m^-1^), and (Salinity-3; 6.5 dS m^-1^) at different growth stages represented by days after transplantation (DAT), as 35 DAT (vegetative stage), 50 DAT (1^st^ cluster flower stage), 60 DAT (3^rd^ cluster flower stage), 75 DAT (fruit development stage) and 85 DAT (fruit ripening stage). Results showed that tomato plants were significantly affected by the imposed salinity treatments. Where, tomato plants treated with salinity-1 was healthier compared to salinily-3 treated plants. This has been concluded from the results of the studied VIs, where the highest mean values of MSI (0.543) and CRSI (0.779) were associated with salinity-3, along with low values of GLI (0.353) and NDNI (0.220), indicating high salinity stress. However, the highest mean values of both NDNI (0.232) and GLI (0.386) were observed for salinity-1, indicated healthy condition. It also proven with the studied photosynthetic parameters, with the highest mean values of P_N_ (9.8 µmol CO_2_ m^-2^ s^-1^),Gs (0.117 mmol H_2_O m^-2^ s^-1^) and Tr (2.236 mmol H_2_O m^-2^ s^-1^) were associated with salinity-1, While the lowest mean values of P_N_ (8.3 µmol CO_2_ m^-2^ s^-1^), Gs (0.102 mmol H_2_O m^-2^ s^-1^) and Tr (1.902 mmol H_2_O m^-2^ s^-1^) were recorded for the plants treated with salinity-1. Moreover, the total tomato fruit yield also decreased significantly at salinity-3 compared to salinity-1.

## Introduction

Tomato (*Solanum lycopersicum* L.) is one of the major fresh and healthy crops worldwide and the second most important agricultural crop after potato [1]. Hernández-Pérezet al. [2] stated that tomato production will become insufficient in the near future, due to increasing demand as a result of population growth and scarcity of land and some abiotic stress factors. Salinity stress is among the most important factors causing a decrease in tomato yield [3]. However, hydroponics, an advanced system for growing plants in greenhouses, has been used to control nutrition, irrigation, and environmental factors [4], control salt concentrations for plants [5], and achieve high productivity and economic returns [6].

Tomatoes are among the most important plants grown under hydroponic systems in greenhouses in Saudi Arabia, as greenhouse tomato cultivation constitutes about 50% of the total tomato production [7]. Saudi Arabia is located in a dry desert environment with very low rainfall, with an average annual rainfall of less than 100 mm and no other water resources for freshwater supplies, such as lakes or rivers [8]. However, using saline water to irrigate crops is an effective solution to overcome the shortage of freshwater [9]. Tomatoes are plants that tolerate moderate salinity, while high salinity concentrations have a negative effect on plant growth and fruit development [10]. Tomatoes can tolerate salinity within an electrical conductivity (EC) range of 1.3 to 6.0 dS m^-1^ [11]. However various studies have reported a reduction in tomato fruit weight of about 10% at an EC of 5.0–6.0 dS m^-1^, and 30% at an EC of 8.0 dS m^-1^ [11]. In fact, the ability of plants to tolerate salinity stress varies based on different growth stages [12], as tolerance to salt stress at a certain stage of plant growth does not mean tolerance at other growth stages [13].

Spectroscopy, which relies on the absorption and reflection of light energy, has become a rapid and non-destructive alternative method for detecting plant stress [11]. Spectral vegetation indices can be described as a mathematical analysis of multiple spectral bands of the electromagnetic spectrum [14]. The spectral vegetation indices can also be used to determine the spatial variability of crops within a field based on crop water availability and variations in soil parameters [15]. The concept of plant spectral reflectance is that healthy plants absorb more light in the visible part of the electromagnetic spectrum with less reflectance, while reflecting more light in the near-infrared part of the spectrum than plants under stress [16]. Roman and Ursu [17], noted that healthy plant leaves absorb about 70–90% of the visible light and reflect green light so that these leaves become visible to the naked eye. However, green light, when absorbed by leaves, activates photosynthesis very efficiently [18]. These different wavelengths of light can directly regulate photosynthesis by affecting stomata, chloroplast development, pigment content, and the activity of photosystems and related enzymes [19]. Hyperspectral measurements have demonstrated the ability to monitor and predict the physiological status of plants under stress [20,21]. The spectral reflectance indices, measured by hyper-spectrometers in the visible and near-infrared ranges, can be used to detect early changes in plants growth due to biotic and abiotic stresses [22]. In this regard, Tola et al. [23], studied the relationship between salinity stress and spectral reflectance, and reported that increasing salinity level was associated with increased spectral reflectance in the Visible, Red-Edge and NIR regions of the electromagnetic spectrum of tomato plants. On the other hand, Zengru et al. [24], demonstrated the possibility of using vegetation indices to estimate plant biomass and photosynthesis. While, Munns [25], reported that plant physiological characteristics, such as leaf photosynthesis, stomatal conductance and water content, are negatively affected by salinity stress. Yang et al. [26] also noticed the negative effect of salt stress on photosynthetic pathways and enzyme activities; which alters the stomatal rhythems. Thus, reduces the rate of transpiration and leads to negative effects on the efficiency of photosynthesis [27]. On the other hand, physiological pathways triggers towards defence mechanism.The energy generated by photosynthesis, was directed towards defense against stress rather than growth [28]. Therefore, the aim of this research was to evaluate the response of hydroponic tomato plants to different levels of salinity during different growth stages, based on spectral vegetation indices and photosynthetic parameters.

## Materials and methods

### Experimental framework

The experimental work was carried out in the greenhouse facility of the Precision Agriculture Research Chair, King Saud University, located in the Riyadh region of Saudi Arabia at 46° 37′ 10″ N & 24° 44′ 12″ E. Tomato seedlings (Valouro-RZ variety) were grown in a greenhouse equipped with a semi-closed hydroponic system and MACQU systems (Geosmart, Athens, Greece) for controlling the indoor climate, irrigation and treatments. The daytime temperature and relative humidity inside the greenhouse ranged from 17°C to 23°C and 55% to 65%, respectively.

Tomato seedlings, after about 4−5 true leaves fully developed, were transplanted into perlite bags in rows 175 cm apart and a distance of 20 cm between plants in the row. The study was conducted during the period from November 15, 2023 to March 15, 2024. Three different salinity levels were applied, where the electric conductivity (EC) of the nutrient solution was 2.5 dS m^-1^ (salinity-1 as a control level), 4.0 dS m^-1^ (salinity-2) and 6.5 dS m^-1^ (salinity-3), and the pH of the nutrient solution was set in the range of 5.5 to 6.5.

### Data collection

The spectral reflectance and leaf photosynthesis of tomato plants grown in the hydroponic greenhouse, under three salinity levels of 2.5, 4.0 and 6.5 dS m^-1^, were measured for three leaves randomly selected from each plant. Measurements were taken at different growth stages represented by plant age in days after transplantation (DAT), denoted as 35 DAT (vegetative stage), 50 DAT (1^st^ cluster flower stage), 60 DAT (3^rd^ cluster flower stage), 75 DAT (fruit development stage) and 85 DAT (fruit ripening stage).

### Spectral vegetation indices

The spectral reflectance of tomato plant leaves was measured using an ASD FieldSpec 3 handheld spectrophotometer (Analytical Spectral Devices Inc., Boulder, CO, USA), with a spectral range of 350–2500 nm. Measurements were performed after calibration of the instrument using a reflectance white reference plate. Plant leaf reflectance was measured, by averaging 15 repeat scans for each tomato plant leaf, using the direct contact probe module. A total of 25 vegetation indices were calculated based on wavelength bands of the reflection of tomato plant leaves. Among the 25 spectral indices, four vegetation indices showed high applicability for evaluating tomato performance under the different salinity levels during different growth stages. Table 1 shows the descriptions and formulas of the four selected vegetation indices.

**Table 1:** Selected spectral vegetation indices used to evaluate tomato plant performance under different salinity levels and growth stages.

Vegetation Indices	Description	Formula	References
MSI	Moisture Stress Index	$\frac{\rho_{1600\;nm}\;}{\rho_{820\;nm}\;}$	[29]
CRSI	Canopy Response Salinity Index	$\sqrt{\frac{\left(NIR\times R)-(G\times B\right)}{\left(NIR\times R)+(G\times B\right)}}$	[30]
NDNI	Normalized Difference Nitrogen Index	$\frac{\log\;(1/\rho_{1510\;nm})-\log\;(1/\rho_{1680\;nm})}{\log(1/\rho_{1510\;nm})+\log\;(1/\rho_{1680\;nm})}$	[31]
GLI	Green Leaf Index	$\frac{(G-R)+(G-B}{(G+R)+(G+B)\;}$	[32]

*ρ: Reflectance band, NIR: Near infrared red, R: Red channel, G: Green channel, B: Blue channel.*

### Leaf photosynthetic parameters

In conjunction with the spectral vegetation indices measurements, photosynthetic parameters of tomato leaves were measured using a portable photosynthesis device (Model: LI-6400XT, Li-Cor Inc., Lincoln, NE, USA). The leaf photosynthetic parameters included Net photosynthesis rate (P_N_, µmol CO_2_ m^-2^ s^-1^), Transpiration rate (Tr, mmol H_2_O m^-2^ s^-1^), Water use efficiency (WUE, µmol CO_2_/ mmol H_2_O) and Total stomatal conductance (Gs, mmol H_2_O m^-2^ s^-1^). The photosynthesis device was calibrated and set to artificial light mode at a flow rate of 500 µmol s^-1^, then measurements were taken from tomato leaves between 8–11 am with an average of 30 readings per leaf sample.

### Tomato fruit yield

Fresh tomato fruits that reached at least 80% red maturity were harvested on a weekly basis. Tomato fruits were weighed using a balance of ± 0.05 accuracy and then the total tomato fruit yield (kg m^-2^) was the cumulative weight of all fruits harvested during the entire period per unit area.

### Statistical analysis

A split plot design analysis of spectral vegetation indices and photosynthetic parameters was conducted using five different growth stages of tomato as main treatments and three different salinity levels as sub-treatments with three replicates. To evaluate the response of tomato plants grown under hydroponic system to different salinity levels, the collected results were subjected to the analysis of variance (ANOVA) using the Statitix 10 software, and the least significant difference (LSD) test was used to compare means at a 5% confidence level.

## Results and discussion

The response of tomato plants grown under a hydroponic system to different salinity levels at different growth stages was studied through selected spectral vegetation indices, photosynthetic parameters and fruit yield. The results of the collected data showed some significant correlations, which will be highlighted in the following sections.

### Spectral vegetation indices

As shown in Table 2, the selected spectral vegetation indices were significantly affected by salinity levels at different tomato growth stages. Both the moisture stress index (MSI) and canopy response salinity index (CRSI) showed a positive linear correlation with salinity levels, as confirmed by Rosadi et al. [33]; that water stress increases with increasing salinity of the nutrient solution. Where the highest mean values of MSI (0.518) and CRSI (0.773) were recorded at salinity-3 (6.5 dS m^-1^), which was significantly different compared the lowest values of MSI (0.483) and CRSI (0.761) recorded at salinity-1 (2.5 dS m^-1^). This could be attributed to the fact that salt stress can lead to reduced root water uptake which in turn leads to increased moisture stress [34], where a higher value of MSI indicates insufficient water content for the plant [14].

**Table 2:** Results of statistical analysis (ANOVA) of spectral vegetation indices for tomato leaves at different salinity levels and growth stages.

Spectral Vegetation Indices	Salinity Levels (S)			LSD	Days After Transplanting (DAT)					LSD
	S-1	S-2	S-3		35	50	60	75	85	
**Moisture Stress Index (MSI)**	**0.483** ^**b**^	**0.501** ^**ab**^	**0.518** ^**a**^	**0.018**	**0.506** ^**ab**^	**0.499** ^**ab**^	**0.500** ^**ab**^	**0.515** ^**a**^	**0.481** ^**b**^	**0.031**
**Canopy Response Salinity Index (CRSI)**	**0.761** ^**b**^	**0.767** ^**ab**^	**0.773** ^**a**^	**0.009**	**0.770** ^**a**^	**0.771** ^**a**^	**0.754** ^**b**^	**0.765** ^**ab**^	**0.775** ^**a**^	**0.015**
**Normalized Difference Nitrogen Index (NDNI)**	**0.225** ^**a**^	**0.223** ^**ab**^	**0.220** ^**b**^	**0.004**	**0.223** ^**b**^	**0.221** ^**b**^	**0.231** ^**a**^	**0.215** ^**c**^	**0.224** ^**b**^	**0.004**
**Green Leaf Index (GLI)**	**0.368** ^**a**^	**0.365** ^**ab**^	**0.353** ^**b**^	**0.015**	**0.363** ^**ab**^	**0.369** ^**a**^	**0.371** ^**a**^	**0.355** ^**ab**^	**0.349** ^**b**^	**0.019**

*Means with different letters indicated significant differences, at the least significant difference (LSD), 5% confidence level.*

Regarding the growth stages, the effect of increasing salt concentration on tomato plants was reflected in the high value of the average moisture stress index (0.515) at 75 days after transplanting (DAT), or fruit development stage, then decreased significantly to 0.481 at 85 DAT (fruit ripening stage). This is attributed to that tomato leaves are exposed to greater water stress during the fruit development stage because of the sensitivity to water stress which occurs mainly during the fruit development stage rather than the flowering and fruiting stages [35,36]. While CRSI values varied up and down during the growth stages until the highest value (0.775) was recorded at 85 DAT (fruit ripening stage), which may be due to the long-term accumulation of salts in the leaves as the tomato growth stage progresses [37]. Also, as shown in Table 2, the Normalized Difference Nitrogen Index (NDNI) and Green Leaf Index (GLI) followed the same trend regarding the effect of salinity levels on tomato plants during the different growth stages. Where nitrogen is closely related to the chlorophyll concentration and indicates the vegetative mass of tomato leaves [38]. The maximum mean values of NDNI (0.225) and GLI (0.368) were recorded at salinity-1 (2.5 dS m^-1^), which were not significantly different compared to salinity-2 (4.0 dS m^-1^). While the NDNI and GLI values decreased significantly by about 2.22% and 4.07%, respectively, at salinity-3 (6.5 dS m^-1^), compared to salinity-1. These results were similar to those of Cakan et al. [39] who reported that salt stress decreased vegetative growth and mineral nutrient content. As for the growth stages, the highest mean values of NDNI (0.231) and GLI (0.371) were recorded at 60 DAT (3^rd^ cluster flower). This may be attributed to the fact that the concentration of nutrients in tomatoes is higher at the fruiting stage compared to the vegetative growth stage. While the lowest mean value of GLI (0.349) was recorded at 85 DAT. The increase of CRSI at this stage indicates that prolonged exposure of tomato leaves to salinity leads to leaf deterioration and impairs plant growth and development [40]. In general, the values of vegetation indices values at 60 DAT were the highest compared to the other growth stages.

**Fig 1:**
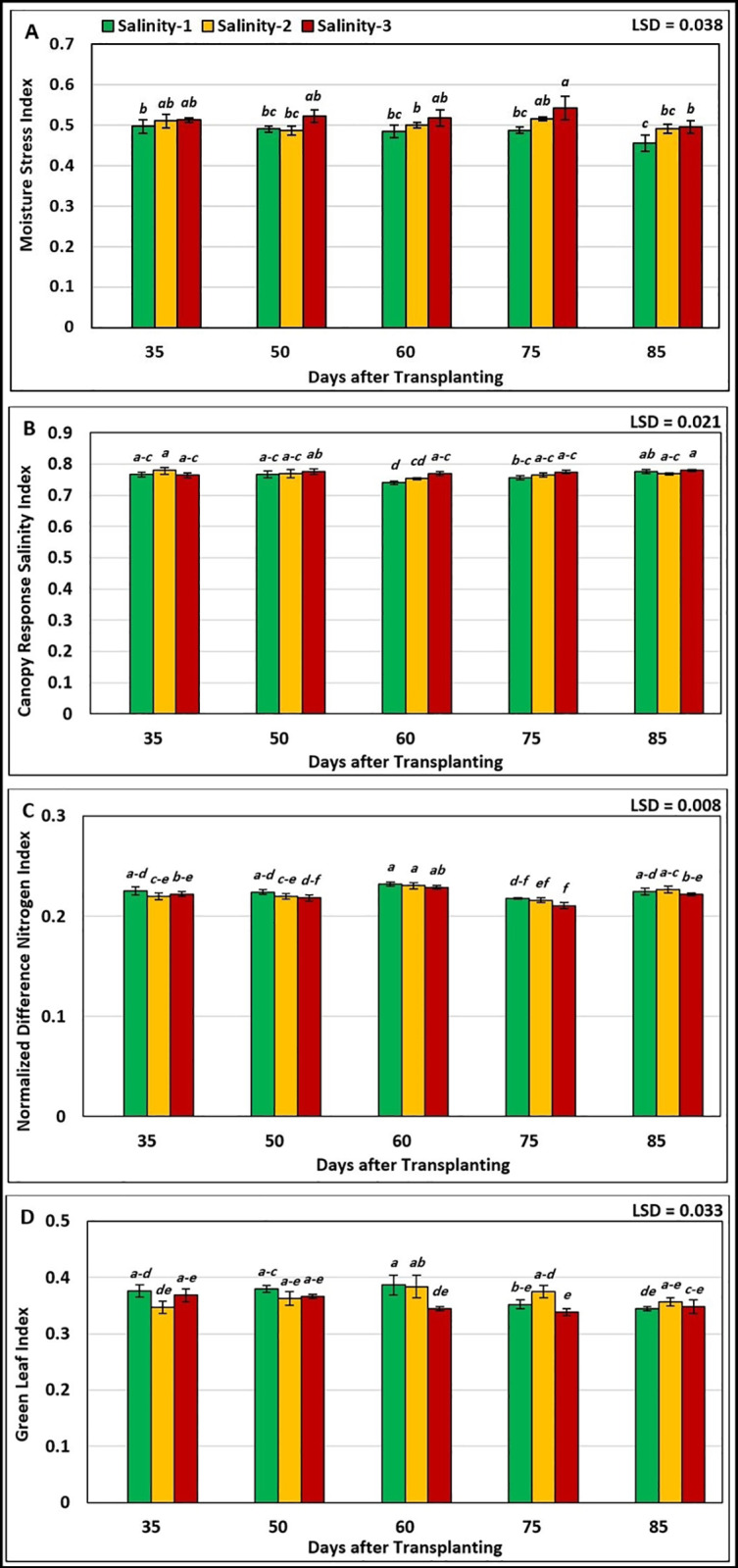
Effect of the interaction between salinity levels and tomato growth stages on the spectral vegetation indices: (A) Moisture Stress Index (MSI), (B) Canopy Response Salinity Index (CRSI), (C) Normalized Difference Nitrogen Index (NDNI), and (D) Green Leaf Index (GLI).

Based on the results of statistical analysis (ANOVA) of the interaction (Fig 1), it was found that there were significant differences between the values of vegetation indices at different salinity levels and growth stages. During all growth stages, tomato plants were under high water stress at the higher salinity level (salinity-3), then the stress decreased at salinity-2, and the lowest water stress was at the lowest salinity level (salinity-1). On the average, the highest mean MSI value (0.543) was recorded at a combination of salinity-3 and 75 DAT (fruit development stage), without significant differences from the lowest value (0.455) recorded at a combination of salinity-1 and 85 DAT (fruit ripening stage). While the highest mean CRSI value (0.779) was recorded at the combination of salinity-3 and 85 DAT. However, tomato plants at the 3^rd^ cluster flower stage (60 DAT) under both salinity-1 and salinity-2 were less affected by salinity, with the lowest mean CRSI values recorded at salinity-1 (0.740) and salinity-2 (0.753). While, the highest mean NDNI value (0.232) and GLI (0.232) were recorded at the combination of salinity-1 and 60 DAT (3^rd^ cluster flower stage), which was not significantly different compared to salinity- 2.

### Leaf photosynthetic parameters

The results of statistical analysis (ANOVA) showed significant differences in the values of tomato leaf photosynthetic parameters among the studied salinity concentrations (salinity-1, salinity-2 and salinity-3) and across the growth stages (Table 3). The mean values of leaf photosynthetic parameters such as P_N_ (9.799 µmol CO_2_ m^-2^ s^-1^), WUE (46.5 µmol CO_2_/ mmol H_2_O), Tr (2.236 mmol H_2_O m^-2^ s^-1^) and Gs (0.117 mmol H_2_O m^-2^ s^-1^) were high in salinity-1 plants, and were not significantly different from those recorded with salinity-2. While the values of photosynthetic parameters recorded with salinity-3 were significantly low compared to salinity-1 and salinity-2, but the WUE showed no significant differences between all salinity levels. These results are consistent with the findings of Habibi et al. [41] who reported an adverse relationship between leaf photosynthetic parameters and salt stress. Saleh et al. [42] also reported that photosynthesis, transpiration rate and stomatal conductance were decreased due to salt stress. Moreover, salt stress is considered one of the negative effects on tomatoes as it reduces the rate of leaf photosynthesis [43], resulting in a decrease in fruit size and overall production.

**Table 3:** Results of statistical analysis (ANOVA) of leaf photosynthetic parameters for tomato leaves at different salinity levels and growth stages.

Leaf Photosynthetic Parameters	Salinity Levels (S)			LSD	Days after Transplanting (DAT)					LSD
	S-1	S-2	S-3		35	50	60	75	85	
**Photosynthetic Rate (P**_**N**_)	**9.798** ^**a**^	**9.309** ^**a**^	**8.332** ^**b**^	**0.962**	**8.304** ^**c**^	**9.703** ^**b**^	**11.825** ^**a**^	**10.915** ^**ab**^	**4.985** ^**d**^	**1.372**
**Water Use Efficiency (WUE)**	**4.382** ^**a**^	**4.276** ^**a**^	**4.383** ^**a**^	**0. 60**	**3.380** ^**c**^	**5.030** ^**b**^	**6.445** ^**a**^	**3.920** ^**b**^	**3.049** ^**c**^	**0. 970**
**Transpiration Rate (Tr)**	**2.236** ^**a**^	**2.177** ^**a**^	**1.902** ^**b**^	**0.215**	**2.464** ^**a**^	**1.976** ^**b**^	**1.835** ^**b**^	**2.615** ^**a**^	**1.636** ^**b**^	**0.459**
**Total Stomatal Conductance (Gs)**	**116** ^**a**^	**118** ^**a**^	**102** ^**b**^	**14**	**146** ^**a**^	**94** ^**b**^	**96** ^**b**^	**139** ^**a**^	**85** ^**b**^	**28**

*Means with different letters showed significant differences, at the least significant difference (LSD), 5% confidence level.*

Although there were no significant differences in the WUE between salinity levels, a slight increase in the WUE was recorded at the high salinity (salinity-3). This result was similar to that of Gharbi et al. [44], that is because the stomatal system being more susceptible to the decreased stomatal conductance under salt stress. However, plant adaptation to salt concentrations occurs through the activation of physiological and biochemical mechanisms, which leads to maximizing WUE [45]. Furthermore, stomatal conductance decreases under salt stress, and thus the plant reduces the opening of the stomata in the leaves to avoid the damage caused by high salt, and to maintain and conserve the leaf water balance [46]. Also, Blatt et al. [47], indicated the ability of plants to prevent or reduce water loss from leaves and improve water use efficiency by modifying stomatal conductance.

To study the effect of different growth stages, a gradual increase in P_N_ and WUE values and a decrease in Tr and Gs values were recorded with plant age up to 60 DAT (Table 3). This result was consistent with Cao et al. [48], who reported that plant growth under high CO_2_ may reduce stomatal conductance and transpiration rate, as an inverse relationship. This in turn will improve the WUE, which ultimately positively affects leaf photosynthesis. Whereas stomata open and close to control the exchange of CO_2_ gas in response to photosynthetic demand [49]. Furthermore, this could be attributed to exposure of tomatoes to high moisture and salinity stresses at early stages (35, 50 DAT), as shown in Table 2, which negatively affected only the photosynthetic rate and water use efficiency resulting from the reduced ability of roots to extract water due to the osmotic stress [50], while all leaf photosynthetic parameters decreased at 85 DAT (fruit ripening stage).

Moreover, the photosynthetic rate (P_N_) was linearly related to the water use efficiency (WUE) at the different growth stages. Consequently, the P_N_ and WUE followed the same trend as the spectral indices of normalized difference nitrogen index (NDNI), and green leaf index (GLI) regarding the effects of salinity levels and some growth stages. The highest average values of P_N_ (11.825 µmol CO_2_ m^-2^ s^-1^) and WUE (6.445 µmol CO_2_/ mmol H_2_O) were recorded at 60 DAT (3^rd^ cluster flower stage), then gradually decreased significantly in other growth stages. Moreover, at 60 DAT, the transpiration rate (Tr) decreased significantly with the increasing WUE, and thus there was a negative relationship between WUE and Tr [51]. On the other hand, the lowest average values of P_N_ (4.985 µmol CO_2_ m^-2^ s^-1^) and WUE (3.049 µmol CO_2_/ mmol H_2_O) were recorded at 85 DAT (fruit ripening stage). Stomatal conductance (Gs) was correlated with the transpiration rate (Tr), as increasing the stomata led to greater transpiration of tomato leaves. However, the Gs and Tr increased at the early growth stage (vegetative stage), and then decreased significantly until the 3^rd^ cluster flower stage.

**Fig 2:**
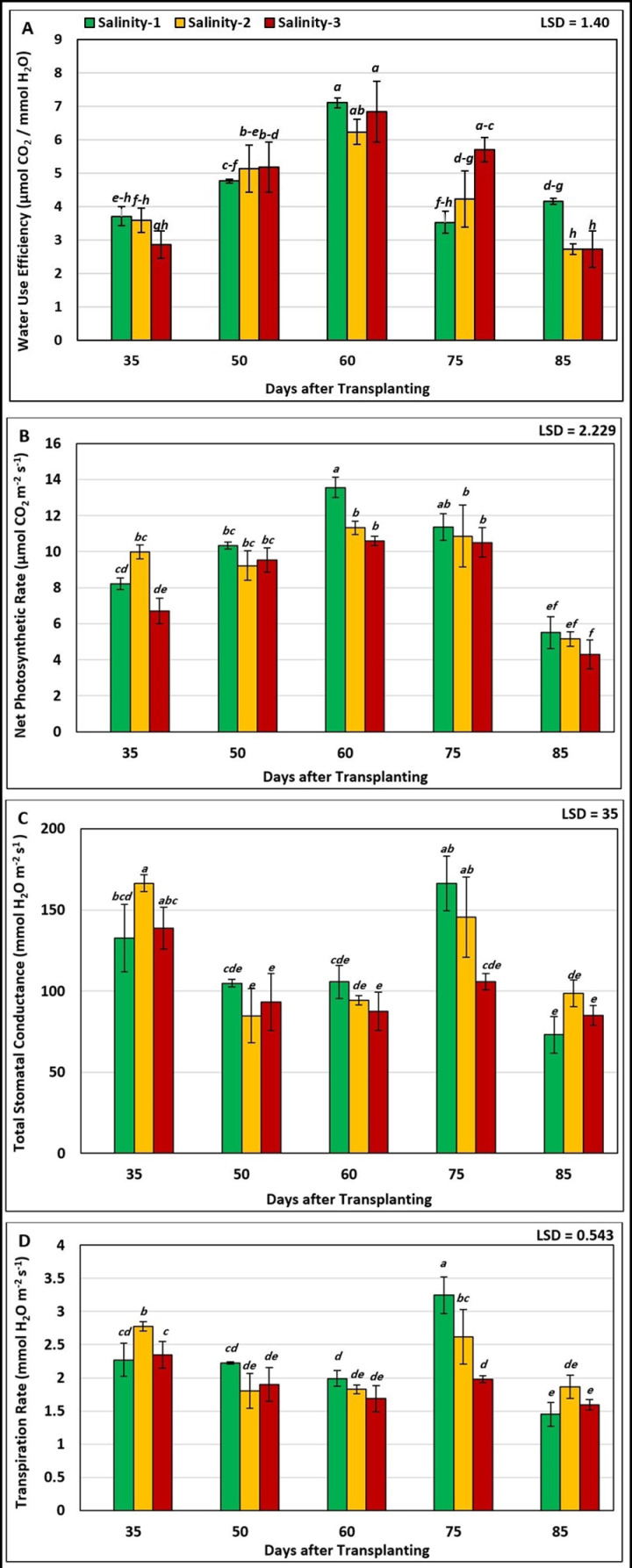
Effect of the interaction between salinity levels and tomato growth stages on leaf photosynthetic parameters: (A) Water Use Efficiency (WUE), (B) Net Photosynthetic Rate (P_N_), (C) Total Stomatal Conductance (Gs), and (D) Transpiration Rate (Tr).

As depicted in Fig 2, the results of statistical analysis (ANOVA) showed significant differences between salinity levels and growth stage in terms of leaf photosynthetic parameters. On the average, the highest mean values of P_N_ (13.550 µmol CO_2_ m^-2^ s^-1^) and WUE (7.110 µmol CO_2_/ mmol H_2_O) were recorded with salinity-1, which were not significantly different from those at salinity-2 and salinity-3 at the 3^rd^ cluster flower stage (60 DAT). While the lowest mean values of P_N_ (4.297 µmol CO^2^ m^-2^ s^-1^) and WUE (2.730 µmol CO_2_/ mmol H_2_O) were recorded with salinity-3 at fruit ripening stage (85 DAT). Conversely, Tr and Gs were most affected by salinity levels at the flowering stage and least affected at the vegetative stage (35 DAT) and fruit development stage (75 DAT). However, the highest mean value of Gs (166 mmol H_2_O m^-2^ s^-1^) was associated with salinity-2 at vegetative stage (35 DAT), which was not significantly different from those at salinity-1 and salinity-2 at the fruit development stage. The highest mean Tr (3.248 mmol H_2_O m^-2^ s^-1^) was associated with salinity-1 at the fruit development stage, while the lowest mean value (1.450 mmol H_2_O m^-2^ s^-1^) was associated with salinity-1 at the fruit ripening stage and was not significantly different from the Tr value (1.828 mmol H_2_O m^-2^ s^-1^) at salinity-2 at the 3^rd^ cluster flower. In general, based on the interaction results, tomato plants at the 3rd cluster flowering stage (60 DAT) were more tolerant to water and salt stress, as reflected in the high values of photosynthetic rate and water use efficiency and the low transpiration rate, especially under salinity-1 and salinity-2.

### Tomato fruit yield

**Fig 3:**
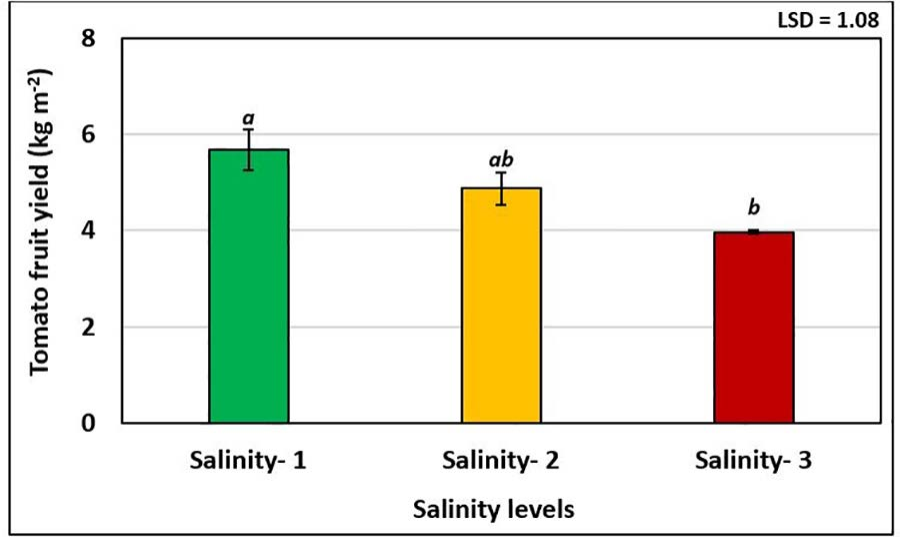
Response of tomato fruit yield to the different salinity levels.

The results of statistical analysis (ANOVA) of the total yield of tomato fruits showed significant changes between different salinity levels (Fig 3). The highest value of tomato fruit yield (5.67 kg m^-2^) was obtained at salinity-1, which was not statistically significant compared to the fruit yield (4.87 kg m^-2^) obtained at salinity-2 and significantly different from the lowest fruit yield (3.97 kg m^-2^) obtained at salinity-3. The total tomato fruit yield was significantly reduced at salinity-3 (6.5 dS m^-1^) by about 29.9% compared to salinity-1. This result was somewhat close to the results of Ignat et al. [11], who reported a yield decresded of about 30% at an EC of 8.0 dS m^-1^. Furthermore, Saleh et al. [42] reported that the vegetative growth of tomato plants (i.e., plant height, number of leaves, and chlorophyll content) was negatively affected by the salinity treatment compared to the non-salinity treatment leading to a decrease in overall yield. However, the tomato fruit yield in terms of salinity levels followed the same trend of the effect of salinity levels on vegetation indices and photosynthetic parameters of tomato leaves. Based on these results, it can be concluded that tomato production in hydroponics can be achieved at salinity levels up to 4.0 dS m^-1^ without affecting the total tomato fruit production.

## Conclusions

This research focused on studying the effects of salinity on the status of hydroponic tomato plants at different growth stages, based on spectral indices (MSI, CRSI, NDNI and GLI), photosynthetic parameters (P_N_, WUE, Tr and Gs), and fruit yield. Measurements were recorded for hydroponic tomato exposed to three salinity levels (2.5, 4.0, and 6.5 dS m^-1^) at different growth stages (35, 50, 60, 75 and 85 DAT). The selected spectral vegetation indices and photosynthetic parameters were negatively affected by increasing salinity levels at different tomato growth stages. Both MSI and CRSI showed a positive linear correlation with salinity levels, this indicates that tomato plants are exposed to more stress as salinity levels increase. No significant differences were recorded between salinity-1 and salinity-2 in the values of vegetation indices, photosynthetic parameters, and fruit yield. Based on the results of this study, it can be concluded that tomatoes grown under hydroponic system can tolerate up to 4 dS m^-1^ without affecting growth and yield.

## Supporting information

S1 Data Manuscript data. (XLSX)

S2 File Statisitcal analysis. (DOCX)
