# Supplementary material for: Use of spectral indices and photosynthetic parameters to evaluate the growth performance of hydroponic tomato at different salinity levels
Source: PLoS One. 2025 Jun 6;20(6):e0325839. doi: 10.1371/journal.pone.0325839 (PMC12143551; doi:10.1371/journal.pone.0325839)
Supplement: S2 File — (DOCX) [file pone.0325839.s002.docx]

**Spectral Vegetation Indices**

Statistix 10.0 (30-day Trial) 9/11/2024, 2:14:40 PM

**Split-plot AOV Table for MSI**

**Source DF SS MS F P**

Rep 3 0.00213 7.095E-04

Date 4 0.00776 1.940E-03 1.55 0.2494

Error Rep*Date 12 0.01499 1.250E-03

Sal 2 0.01254 6.272E-03 8.41 0.0013

Date*Sal 8 0.00353 4.410E-04 0.59 0.7774

Error Rep*Date*Sal 30 0.02238 7.461E-04

Total 59 0.06334

Grand Mean 0.5007

CV(Rep*Date) 7.06

CV(Rep*Date*Sal) 5.46

**Split-plot AOV Table for NDNI**

**Source DF SS MS F P**

Rep 3 3.303E-05 1.101E-05

Date 4 1.566E-03 3.915E-04 16.99 0.0001

Error Rep*Date 12 2.765E-04 2.304E-05

Sal 2 1.913E-04 9.567E-05 2.76 0.0796

Date*Sal 8 1.225E-04 1.531E-05 0.44 0.8864

Error Rep*Date*Sal 30 1.041E-03 3.470E-05

Total 59 3.230E-03

Grand Mean 0.2226

CV(Rep*Date) 2.16

CV(Rep*Date*Sal) 2.65

**Split-plot AOV Table for GLI**

**Source DF SS MS F P**

Rep 3 0.00017 5.820E-05

Date 4 0.00423 1.058E-03 2.24 0.1257

Error Rep*Date 12 0.00567 4.726E-04

Sal 2 0.00245 1.223E-03 2.42 0.1058

Date*Sal 8 0.00743 9.289E-04 1.84 0.1083

Error Rep*Date*Sal 30 0.01515 5.048E-04

Total 59 0.03510

Grand Mean 0.3618

CV(Rep*Date) 6.01

CV(Rep*Date*Sal) 6.21

**Split-plot AOV Table for CRSI**

**Source DF SS MS F P**

Rep 3 0.00117 3.904E-04

Date 4 0.00307 7.687E-04 2.52 0.0961

Error Rep*Date 12 0.00366 3.047E-04

Sal 2 0.00139 6.963E-04 3.62 0.0391

Date*Sal 8 0.00203 2.532E-04 1.32 0.2739

Error Rep*Date*Sal 30 0.00577 1.925E-04

Total 59 0.01709

Grand Mean 0.7671

CV(Rep*Date) 2.28

CV(Rep*Date*Sal) 1.81

Statistix 10.0 (30-day Trial) 9/11/2024, 2:15:37 PM

**LSD All-Pairwise Comparisons Test of MSI for Rep**

**Rep Mean Homogeneous Groups**

2 0.5095 A

4 0.5029 A

3 0.4954 A

1 0.4951 A

Alpha 0.05 Standard Error for Comparison 0.0129

Critical T Value 2.179 Critical Value for Comparison 0.0281

There are no significant pairwise differences among the means.

**LSD All-Pairwise Comparisons Test of MSI for Date**

**Date Mean Homogeneous Groups**

4 0.5156 A

1 0.5069 AB

3 0.5003 AB

2 0.4997 AB

5 0.4811 B

Alpha 0.05 Standard Error for Comparison 0.0144

Critical T Value 2.179 Critical Value for Comparison 0.0314

There are 2 groups (A and B) in which the means

are not significantly different from one another.

**LSD All-Pairwise Comparisons Test of MSI for Sal**

**Sal Mean Homogeneous Groups**

3 0.5184 A

2 0.5009 A

1 0.4830 B

Alpha 0.05 Standard Error for Comparison 8.638E-03

Critical T Value 2.042 Critical Value for Comparison 0.0176

There are 2 groups (A and B) in which the means

are not significantly different from one another.

**LSD All-Pairwise Comparisons Test of MSI for Date*Sal**

**Date Sal Mean 1,1 1,2 1,3 2,1 2,2 2,3**

1 1 0.4975

1 2 0.5108 0.0133

1 3 0.5126 0.0151 0.0017

2 1 0.4902 0.0073 0.0206 0.0223

2 2 0.4865 0.0110 0.0243 0.0261 0.0037

2 3 0.5224 0.0250 0.0116 0.0099 0.0322 0.0359

3 1 0.4838 0.0137 0.0271 0.0288 0.0064 0.0027 0.0387

3 2 0.4995 0.0020 0.0113 0.0131 0.0093 0.0130 0.0229

3 3 0.5178 0.0203 0.0069 0.0052 0.0275 0.0313 0.0047

4 1 0.4878 0.0097 0.0230 0.0248 0.0024 0.0013 0.0347

4 2 0.5158 0.0183 0.0049 0.0032 0.0255 0.0293 0.0067

4 3 0.5432 0.0457* 0.0324 0.0306 0.0530* 0.0567* 0.0208

5 1 0.4555 0.0419 0.0553* 0.0570* 0.0347 0.0310 0.0669*

5 2 0.4918 0.0057 0.0191 0.0208 0.0016 0.0053 0.0307

5 3 0.4959 0.0016 0.0149 0.0167 0.0057 0.0094 0.0265

**Date Sal Mean 3,1 3,2 3,3 4,1 4,2 4,3**

3 2 0.4995 0.0157

3 3 0.5178 0.0340 0.0183

4 1 0.4878 0.0040 0.0117 0.0300

4 2 0.5158 0.0320 0.0163 0.0020 0.0280

4 3 0.5432 0.0594* 0.0437 0.0254 0.0554* 0.0274

5 1 0.4555 0.0282 0.0439 0.0622* 0.0322 0.0602* 0.0877*

5 2 0.4918 0.0080 0.0077 0.0260 0.0040 0.0240 0.0514*

5 3 0.4959 0.0121 0.0036 0.0219 0.0081 0.0199 0.0473*

**Date Sal Mean 5,1 5,2**

5 2 0.4918 0.0362

5 3 0.4959 0.0404* 0.0041

Comparisons of means for the same level of Date

Alpha 0.05 Standard Error for Comparison 0.0193

Critical T Value 2.042 Critical Value for Comparison 0.0394

Error term used: Rep*Date*Sal, 30 DF

Comparisons of means for different levels of Date

Alpha 0.05 Standard Error for Comparison 0.0214

Critical T Value 2.104 Critical Value for Comparison 0.0450

Error terms used: Rep*Date and Rep*Date*Sal

The homogeneous group format can't be used

because of the pattern of significant differences.

**LSD All-Pairwise Comparisons Test of NDNI for Rep**

**Rep Mean Homogeneous Groups**

1 0.2231 A

4 0.2231 A

3 0.2230 A

2 0.2213 A

Alpha 0.05 Standard Error for Comparison 1.753E-03

Critical T Value 2.179 Critical Value for Comparison 3.819E-03

There are no significant pairwise differences among the means.

**LSD All-Pairwise Comparisons Test of NDNI for Date**

**Date Mean Homogeneous Groups**

3 0.2306 A

5 0.2244 B

1 0.2225 B

2 0.2208 B

4 0.2148 C

Alpha 0.05 Standard Error for Comparison 1.960E-03

Critical T Value 2.179 Critical Value for Comparison 4.270E-03

There are 3 groups (A, B, etc.) in which the means

are not significantly different from one another.

**LSD All-Pairwise Comparisons Test of NDNI for Sal**

**Sal Mean Homogeneous Groups**

1 0.2248 A

2 0.2226 AB

3 0.2204 B

Alpha 0.05 Standard Error for Comparison 1.863E-03

Critical T Value 2.042 Critical Value for Comparison 3.804E-03

There are 2 groups (A and B) in which the means

are not significantly different from one another.

**LSD All-Pairwise Comparisons Test of NDNI for Date*Sal**

**Date Sal Mean Homogeneous Groups**

3 1 0.2321 A

3 2 0.2307 A

3 3 0.2290 AB

5 2 0.2268 ABC

1 1 0.2253 ABCD

5 1 0.2245 ABCD

2 1 0.2243 ABCD

1 3 0.2222 BCDE

5 3 0.2219 BCDE

1 2 0.2199 CDE

2 2 0.2198 CDE

2 3 0.2184 DEF

4 1 0.2179 DEF

4 2 0.2159 EF

4 3 0.2107 F

Comparisons of means for the same level of Date

Alpha 0.05 Standard Error for Comparison 4.165E-03

Critical T Value 2.042 Critical Value for Comparison 8.506E-03

Error term used: Rep*Date*Sal, 30 DF

Comparisons of means for different levels of Date

Alpha 0.05 Standard Error for Comparison 3.925E-03

Critical T Value 2.076 Critical Value for Comparison 8.150E-03

Error terms used: Rep*Date and Rep*Date*Sal

There are 6 groups (A, B, etc.) in which the means

are not significantly different from one another.

**LSD All-Pairwise Comparisons Test of GLI for Rep**

**Rep Mean Homogeneous Groups**

3 0.3640 A

1 0.3624 A

2 0.3617 A

4 0.3593 A

Alpha 0.05 Standard Error for Comparison 7.938E-03

Critical T Value 2.179 Critical Value for Comparison 0.0173

There are no significant pairwise differences among the means.

**LSD All-Pairwise Comparisons Test of GLI for Date**

**Date Mean Homogeneous Groups**

3 0.3714 A

2 0.3697 A

1 0.3635 AB

4 0.3549 AB

5 0.3496 B

Alpha 0.05 Standard Error for Comparison 8.875E-03

Critical T Value 2.179 Critical Value for Comparison 0.0193

There are 2 groups (A and B) in which the means

are not significantly different from one another.

**LSD All-Pairwise Comparisons Test of GLI for Sal**

**Sal Mean Homogeneous Groups**

1 0.3678 A

2 0.3648 AB

3 0.3530 B

Alpha 0.05 Standard Error for Comparison 7.105E-03

Critical T Value 2.042 Critical Value for Comparison 0.0145

There are 2 groups (A and B) in which the means

are not significantly different from one another.

**LSD All-Pairwise Comparisons Test of GLI for Date*Sal**

**Date Sal Mean Homogeneous Groups**

3 1 0.3863 A

3 2 0.3835 AB

2 1 0.3800 ABC

1 1 0.3759 ABCD

4 2 0.3746 ABCD

1 3 0.3681 ABCDE

2 3 0.3661 ABCDE

2 2 0.3629 ABCDE

5 2 0.3562 ABCDE

4 1 0.3521 BCDE

5 3 0.3480 CDE

1 2 0.3465 DE

5 1 0.3447 DE

3 3 0.3445 DE

4 3 0.3381 E

Comparisons of means for the same level of Date

Alpha 0.05 Standard Error for Comparison 0.0159

Critical T Value 2.042 Critical Value for Comparison 0.0324

Error term used: Rep*Date*Sal, 30 DF

Comparisons of means for different levels of Date

Alpha 0.05 Standard Error for Comparison 0.0157

Critical T Value 2.086 Critical Value for Comparison 0.0328

Error terms used: Rep*Date and Rep*Date*Sal

There are 5 groups (A, B, etc.) in which the means

are not significantly different from one another.

**LSD All-Pairwise Comparisons Test of CRSI for Rep**

**Rep Mean Homogeneous Groups**

2 0.7735 A

3 0.7675 A

4 0.7665 A

1 0.7611 A

Alpha 0.05 Standard Error for Comparison 6.374E-03

Critical T Value 2.179 Critical Value for Comparison 0.0139

There are no significant pairwise differences among the means.

**LSD All-Pairwise Comparisons Test of CRSI for Date**

**Date Mean Homogeneous Groups**

5 0.7750 A

2 0.7711 A

1 0.7700 A

4 0.7654 AB

3 0.7542 B

Alpha 0.05 Standard Error for Comparison 7.126E-03

Critical T Value 2.179 Critical Value for Comparison 0.0155

There are 2 groups (A and B) in which the means

are not significantly different from one another.

**LSD All-Pairwise Comparisons Test of CRSI for Sal**

**Sal Mean Homogeneous Groups**

3 0.7730 A

2 0.7672 AB

1 0.7612 B

Alpha 0.05 Standard Error for Comparison 4.387E-03

Critical T Value 2.042 Critical Value for Comparison 8.960E-03

There are 2 groups (A and B) in which the means

are not significantly different from one another.

**LSD All-Pairwise Comparisons Test of CRSI for Date*Sal**

**Date Sal Mean Homogeneous Groups**

5 3 0.7798 A

1 2 0.7793 A

5 1 0.7767 AB

2 3 0.7765 AB

4 3 0.7748 ABC

2 2 0.7695 ABC

3 3 0.7694 ABC

5 2 0.7684 ABC

2 1 0.7672 ABC

1 1 0.7662 ABC

4 2 0.7656 ABC

1 3 0.7647 ABC

4 1 0.7559 BCD

3 2 0.7530 CD

3 1 0.7401 D

Comparisons of means for the same level of Date

Alpha 0.05 Standard Error for Comparison 9.810E-03

Critical T Value 2.042 Critical Value for Comparison 0.0200

Error term used: Rep*Date*Sal, 30 DF

Comparisons of means for different levels of Date

Alpha 0.05 Standard Error for Comparison 0.0107

Critical T Value 2.103 Critical Value for Comparison 0.0225

Error terms used: Rep*Date and Rep*Date*Sal

There are 4 groups (A, B, etc.) in which the means

are not significantly different from one another.

Statistix 10.0 (30-day Trial) 9/11/2024, 2:16:01 PM

**Means of MSI for Rep**

**Rep Mean**

1 0.4951

2 0.5095

3 0.4954

4 0.5029

Observations per Mean 15

Standard Error of a Mean 9.127E-03

Std Error (Diff of 2 Means) 0.0129

Error term used: Rep*Date, 12 DF

**Means of MSI for Date**

**Date Mean**

1 0.5069

2 0.4997

3 0.5003

4 0.5156

5 0.4811

Observations per Mean 12

Standard Error of a Mean 0.0102

Std Error (Diff of 2 Means) 0.0144

Error term used: Rep*Date, 12 DF

**Means of MSI for Sal**

**Sal Mean**

1 0.4830

2 0.5009

3 0.5184

Observations per Mean 20

Standard Error of a Mean 6.108E-03

Std Error (Diff of 2 Means) 8.638E-03

Error term used: Rep*Date*Sal, 30 DF

**Means of MSI for Date*Sal**

**Date Sal Mean**

1 1 0.4975

1 2 0.5108

1 3 0.5126

2 1 0.4902

2 2 0.4865

2 3 0.5224

3 1 0.4838

3 2 0.4995

3 3 0.5178

4 1 0.4878

4 2 0.5158

4 3 0.5432

5 1 0.4555

5 2 0.4918

5 3 0.4959

Observations per Mean 4

Standard Error of a Mean 0.0137

Error term used: Rep*Date*Sal, 30 DF

**Means of NDWI for Rep**

**Rep Mean**

1 0.0706

2 0.0679

3 0.0706

4 0.0706

Observations per Mean 15

Standard Error of a Mean 1.948E-03

Std Error (Diff of 2 Means) 2.756E-03

Error term used: Rep*Date, 12 DF

**Means of NDWI for Date**

**Date Mean**

1 0.0657

2 0.0733

3 0.0643

4 0.0665

5 0.0798

Observations per Mean 12

Standard Error of a Mean 2.178E-03

Std Error (Diff of 2 Means) 3.081E-03

Error term used: Rep*Date, 12 DF

**Means of NDWI for Sal**

**Sal Mean**

1 0.0736

2 0.0684

3 0.0678

Observations per Mean 20

Standard Error of a Mean 1.427E-03

Std Error (Diff of 2 Means) 2.018E-03

Error term used: Rep*Date*Sal, 30 DF

**Means of NDWI for Date*Sal**

**Date Sal Mean**

1 1 0.0667

1 2 0.0657

1 3 0.0646

2 1 0.0747

2 2 0.0744

2 3 0.0707

3 1 0.0666

3 2 0.0632

3 3 0.0630

4 1 0.0724

4 2 0.0634

4 3 0.0638

5 1 0.0873

5 2 0.0751

5 3 0.0771

Observations per Mean 4

Standard Error of a Mean 3.190E-03

Error term used: Rep*Date*Sal, 30 DF

**Means of PSRI for Rep**

**Rep Mean**

1 -0.0601

2 -0.0551

3 -0.0571

4 -0.0587

Observations per Mean 15

Standard Error of a Mean 1.598E-03

Std Error (Diff of 2 Means) 2.260E-03

Error term used: Rep*Date, 12 DF

**Means of PSRI for Date**

**Date Mean**

1 -0.0570

2 -0.0546

3 -0.0622

4 -0.0604

5 -0.0547

Observations per Mean 12

Standard Error of a Mean 1.786E-03

Std Error (Diff of 2 Means) 2.526E-03

Error term used: Rep*Date, 12 DF

**Means of PSRI for Sal**

**Sal Mean**

1 -0.0596

2 -0.0579

3 -0.0558

Observations per Mean 20

Standard Error of a Mean 1.240E-03

Std Error (Diff of 2 Means) 1.753E-03

Error term used: Rep*Date*Sal, 30 DF

**Means of PSRI for Date*Sal**

**Date Sal Mean**

1 1 -0.0569

1 2 -0.0559

1 3 -0.0583

2 1 -0.0546

2 2 -0.0564

2 3 -0.0526

3 1 -0.0672

3 2 -0.0616

3 3 -0.0578

4 1 -0.0652

4 2 -0.0583

4 3 -0.0577

5 1 -0.0540

5 2 -0.0573

5 3 -0.0526

Observations per Mean 4

Standard Error of a Mean 2.772E-03

Error term used: Rep*Date*Sal, 30 DF

**Means of NDNI for Rep**

**Rep Mean**

1 0.2231

2 0.2213

3 0.2230

4 0.2231

Observations per Mean 15

Standard Error of a Mean 1.239E-03

Std Error (Diff of 2 Means) 1.753E-03

Error term used: Rep*Date, 12 DF

**Means of NDNI for Date**

**Date Mean**

1 0.2225

2 0.2208

3 0.2306

4 0.2148

5 0.2244

Observations per Mean 12

Standard Error of a Mean 1.386E-03

Std Error (Diff of 2 Means) 1.960E-03

Error term used: Rep*Date, 12 DF

**Means of NDNI for Sal**

**Sal Mean**

1 0.2248

2 0.2226

3 0.2204

Observations per Mean 20

Standard Error of a Mean 1.317E-03

Std Error (Diff of 2 Means) 1.863E-03

Error term used: Rep*Date*Sal, 30 DF

**Means of NDNI for Date*Sal**

**Date Sal Mean**

1 1 0.2253

1 2 0.2199

1 3 0.2222

2 1 0.2243

2 2 0.2198

2 3 0.2184

3 1 0.2321

3 2 0.2307

3 3 0.2290

4 1 0.2179

4 2 0.2159

4 3 0.2107

5 1 0.2245

5 2 0.2268

5 3 0.2219

Observations per Mean 4

Standard Error of a Mean 2.945E-03

Error term used: Rep*Date*Sal, 30 DF

**Means of GLI for Rep**

**Rep Mean**

1 0.3624

2 0.3617

3 0.3640

4 0.3593

Observations per Mean 15

Standard Error of a Mean 5.613E-03

Std Error (Diff of 2 Means) 7.938E-03

Error term used: Rep*Date, 12 DF

**Means of GLI for Date**

**Date Mean**

1 0.3635

2 0.3697

3 0.3714

4 0.3549

5 0.3496

Observations per Mean 12

Standard Error of a Mean 6.276E-03

Std Error (Diff of 2 Means) 8.875E-03

Error term used: Rep*Date, 12 DF

**Means of GLI for Sal**

**Sal Mean**

1 0.3678

2 0.3648

3 0.3530

Observations per Mean 20

Standard Error of a Mean 5.024E-03

Std Error (Diff of 2 Means) 7.105E-03

Error term used: Rep*Date*Sal, 30 DF

**Means of GLI for Date*Sal**

**Date Sal Mean**

1 1 0.3759

1 2 0.3465

1 3 0.3681

2 1 0.3800

2 2 0.3629

2 3 0.3661

3 1 0.3863

3 2 0.3835

3 3 0.3445

4 1 0.3521

4 2 0.3746

4 3 0.3381

5 1 0.3447

5 2 0.3562

5 3 0.3480

Observations per Mean 4

Standard Error of a Mean 0.0112

Error term used: Rep*Date*Sal, 30 DF

**Means of CRSI for Rep**

**Rep Mean**

1 0.7611

2 0.7735

3 0.7675

4 0.7665

Observations per Mean 15

Standard Error of a Mean 4.507E-03

Std Error (Diff of 2 Means) 6.374E-03

Error term used: Rep*Date, 12 DF

**Means of CRSI for Date**

**Date Mean**

1 0.7700

2 0.7711

3 0.7542

4 0.7654

5 0.7750

Observations per Mean 12

Standard Error of a Mean 5.039E-03

Std Error (Diff of 2 Means) 7.126E-03

Error term used: Rep*Date, 12 DF

**Means of CRSI for Sal**

**Sal Mean**

1 0.7612

2 0.7672

3 0.7730

Observations per Mean 20

Standard Error of a Mean 3.102E-03

Std Error (Diff of 2 Means) 4.387E-03

Error term used: Rep*Date*Sal, 30 DF

**Means of CRSI for Date*Sal**

**Date Sal Mean**

1 1 0.7662

1 2 0.7793

1 3 0.7647

2 1 0.7672

2 2 0.7695

2 3 0.7765

3 1 0.7401

3 2 0.7530

3 3 0.7694

4 1 0.7559

4 2 0.7656

4 3 0.7748

5 1 0.7767

5 2 0.7684

5 3 0.7798

Observations per Mean 4

Standard Error of a Mean 6.937E-03

Error term used: Rep*Date*Sal, 30 DF

**Photosynthesis Parameters**

Statistix 10.0 (30-day Trial) 9/11/2024, 2:17:00 PM

**Split-plot AOV Table for WUE**

**Source DF SS MS F P**

Rep 3 0.04394 0.01465

Date 4 0.97786 0.24446 20.70 0.0000

Error Rep*Date 12 0.14173 0.01181

Sal 2 0.00991 0.00495 0.57 0.5697

Date*Sal 8 0.18103 0.02263 2.62 0.0265

Error Rep*Date*Sal 30 0.25923 0.00864

Total 59 1.61370

Grand Mean 0.4567

CV(Rep*Date) 23.80

CV(Rep*Date*Sal) 20.35

**Split-plot AOV Table for Photo**

**Source DF SS MS F P**

Rep 3 3.741 1.2468

Date 4 343.646 85.9116 36.11 0.0000

Error Rep*Date 12 28.552 2.3793

Sal 2 22.312 11.1562 5.03 0.0131

Date*Sal 8 25.234 3.1542 1.42 0.2284

Error Rep*Date*Sal 30 66.594 2.2198

Total 59 490.078

Grand Mean 9.1466

CV(Rep*Date) 16.86

CV(Rep*Date*Sal) 16.29

**Split-plot AOV Table for Trmmol**

**Source DF SS MS F P**

Rep 3 0.8595 0.28651

Date 4 8.3759 2.09397 7.85 0.0024

Error Rep*Date 12 3.2022 0.26685

Sal 2 1.2769 0.63844 5.75 0.0077

Date*Sal 8 3.4612 0.43264 3.90 0.0030

Error Rep*Date*Sal 30 3.3302 0.11101

Total 59 20.5058

Grand Mean 2.1052

CV(Rep*Date) 24.54

CV(Rep*Date*Sal) 15.83

**Split-plot AOV Table for CndTotal**

**Source DF SS MS F P**

Rep 3 0.00465 1.550E-03

Date 4 0.03806 9.514E-03 9.28 0.0012

Error Rep*Date 12 0.01230 1.025E-03

Sal 2 0.00306 1.532E-03 3.18 0.0560

Date*Sal 8 0.00986 1.232E-03 2.56 0.0296

Error Rep*Date*Sal 30 0.01446 4.819E-04

Total 59 0.08239

Grand Mean 0.1122

CV(Rep*Date) 28.54

CV(Rep*Date*Sal) 19.57

Statistix 10.0 (30-day Trial) 9/11/2024, 2:18:02 PM

**LSD All-Pairwise Comparisons Test of WUE for Rep**

**Rep Mean Homogeneous Groups**

1 0.4962 A

4 0.4642 A

3 0.4436 A

2 0.4229 A

Alpha 0.05 Standard Error for Comparison 0.0397

Critical T Value 2.179 Critical Value for Comparison 0.0865

There are no significant pairwise differences among the means.

**LSD All-Pairwise Comparisons Test of WUE for Date**

**Date Mean Homogeneous Groups**

3 0.6731 A

2 0.5026 B

4 0.4488 B

1 0.3386 C

5 0.3205 C

Alpha 0.05 Standard Error for Comparison 0.0444

Critical T Value 2.179 Critical Value for Comparison 0.0967

There are 3 groups (A, B, etc.) in which the means

are not significantly different from one another.

**LSD All-Pairwise Comparisons Test of WUE for Sal**

**Sal Mean Homogeneous Groups**

3 0.4662 A

1 0.4654 A

2 0.4385 A

Alpha 0.05 Standard Error for Comparison 0.0294

Critical T Value 2.042 Critical Value for Comparison 0.0600

There are no significant pairwise differences among the means.

**LSD All-Pairwise Comparisons Test of WUE for Date*Sal**

**Date Sal Mean Homogeneous Groups**

3 1 0.7105 A

3 3 0.6850 A

3 2 0.6237 AB

4 3 0.5705 ABC

2 3 0.5178 BCD

2 2 0.5139 BCDE

2 1 0.4762 CDEF

4 2 0.4226 DEFG

5 1 0.4161 DEFG

1 1 0.3709 EFGH

1 2 0.3596 FGH

4 1 0.3531 FGH

1 3 0.2852 GH

5 2 0.2729 H

5 3 0.2725 H

Comparisons of means for the same level of Date

Alpha 0.05 Standard Error for Comparison 0.0657

Critical T Value 2.042 Critical Value for Comparison 0.1342

Error term used: Rep*Date*Sal, 30 DF

Comparisons of means for different levels of Date

Alpha 0.05 Standard Error for Comparison 0.0696

Critical T Value 2.098 Critical Value for Comparison 0.1461

Error terms used: Rep*Date and Rep*Date*Sal

There are 8 groups (A, B, etc.) in which the means

are not significantly different from one another.

**LSD All-Pairwise Comparisons Test of Photo for Rep**

**Rep Mean Homogeneous Groups**

3 9.4947 A

2 9.1819 A

4 9.1184 A

1 8.7914 A

Alpha 0.05 Standard Error for Comparison 0.5632

Critical T Value 2.179 Critical Value for Comparison 1.2272

There are no significant pairwise differences among the means.

**LSD All-Pairwise Comparisons Test of Photo for Date**

**Date Mean Homogeneous Groups**

3 11.825 A

4 10.915 AB

2 9.703 B

1 8.304 C

5 4.985 D

Alpha 0.05 Standard Error for Comparison 0.6297

Critical T Value 2.179 Critical Value for Comparison 1.3721

There are 4 groups (A, B, etc.) in which the means

are not significantly different from one another.

**LSD All-Pairwise Comparisons Test of Photo for Sal**

**Sal Mean Homogeneous Groups**

1 9.7986 A

2 9.3095 A

3 8.3317 B

Alpha 0.05 Standard Error for Comparison 0.4711

Critical T Value 2.042 Critical Value for Comparison 0.9622

There are 2 groups (A and B) in which the means

are not significantly different from one another.

**LSD All-Pairwise Comparisons Test of Photo for Date*Sal**

**Date Sal Mean Homogeneous Groups**

3 1 13.553 A

4 1 11.368 AB

3 2 11.324 B

4 2 10.867 B

3 3 10.598 B

4 3 10.510 B

2 1 10.350 BC

1 2 9.978 BC

2 3 9.535 BC

2 2 9.224 BC

1 1 8.217 CD

1 3 6.718 DE

5 1 5.505 EF

5 2 5.154 EF

5 3 4.297 F

Comparisons of means for the same level of Date

Alpha 0.05 Standard Error for Comparison 1.0535

Critical T Value 2.042 Critical Value for Comparison 2.1516

Error term used: Rep*Date*Sal, 30 DF

Comparisons of means for different levels of Date

Alpha 0.05 Standard Error for Comparison 1.0661

Critical T Value 2.090 Critical Value for Comparison 2.2280

Error terms used: Rep*Date and Rep*Date*Sal

There are 6 groups (A, B, etc.) in which the means

are not significantly different from one another.

**LSD All-Pairwise Comparisons Test of Trmmol for Rep**

**Rep Mean Homogeneous Groups**

2 2.2534 A

3 2.1709 A

4 2.0620 A

1 1.9344 A

Alpha 0.05 Standard Error for Comparison 0.1886

Critical T Value 2.179 Critical Value for Comparison 0.4110

There are no significant pairwise differences among the means.

**LSD All-Pairwise Comparisons Test of Trmmol for Date**

**Date Mean Homogeneous Groups**

4 2.6149 A

1 2.4638 A

2 1.9758 B

3 1.8350 B

5 1.6363 B

Alpha 0.05 Standard Error for Comparison 0.2109

Critical T Value 2.179 Critical Value for Comparison 0.4595

There are 2 groups (A and B) in which the means

are not significantly different from one another.

**LSD All-Pairwise Comparisons Test of Trmmol for Sal**

**Sal Mean Homogeneous Groups**

1 2.2365 A

2 2.1773 A

3 1.9017 B

Alpha 0.05 Standard Error for Comparison 0.1054

Critical T Value 2.042 Critical Value for Comparison 0.2152

There are 2 groups (A and B) in which the means

are not significantly different from one another.

**LSD All-Pairwise Comparisons Test of Trmmol for Date*Sal**

**Date Sal Mean 1,1 1,2 1,3 2,1 2,2 2,3**

1 1 2.2700

1 2 2.7739 0.5039*

1 3 2.3474 0.0774 0.4265

2 1 2.2236 0.0464 0.5503 0.1238

2 2 1.8014 0.4686 0.9725* 0.5460 0.4222

2 3 1.9023 0.3677 0.8716* 0.4451 0.3213 0.1010

3 1 1.9909 0.2791 0.7831* 0.3566 0.2328 0.1895 0.0885

3 2 1.8281 0.4419 0.9458* 0.5193 0.3955 0.0267 0.0742

3 3 1.6860 0.5840 1.0879* 0.6614* 0.5376 0.1153 0.2163

4 1 3.2479 0.9778* 0.4739 0.9004* 1.0242* 1.4465* 1.3455*

4 2 2.6172 0.3472 0.1567 0.2698 0.3936 0.8158* 0.7149*

4 3 1.9797 0.2903 0.7942* 0.3677 0.2439 0.1783 0.0774

5 1 1.4500 0.8200* 1.3239* 0.8975* 0.7737* 0.3514 0.4524

5 2 1.8660 0.4040 0.9079* 0.4814 0.3576 0.0647 0.0363

5 3 1.5930 0.6770* 1.1809* 0.7544* 0.6306* 0.2084 0.3093

**Date Sal Mean 3,1 3,2 3,3 4,1 4,2 4,3**

3 2 1.8281 0.1627

3 3 1.6860 0.3048 0.1421

4 1 3.2479 1.2570* 1.4197* 1.5618*

4 2 2.6172 0.6264* 0.7891* 0.9312* 0.6306*

4 3 1.9797 0.0112 0.1516 0.2937 1.2681* 0.6375*

5 1 1.4500 0.5409 0.3781 0.2361 1.7979* 1.1673* 0.5297

5 2 1.8660 0.1248 0.0379 0.1800 1.3818* 0.7512* 0.1137

5 3 1.5930 0.3979 0.2351 0.0930 1.6549* 1.0242* 0.3867

**Date Sal Mean 5,1 5,2**

5 2 1.8660 0.4161

5 3 1.5930 0.1430 0.2730

Comparisons of means for the same level of Date

Alpha 0.05 Standard Error for Comparison 0.2356

Critical T Value 2.042 Critical Value for Comparison 0.4811

Error term used: Rep*Date*Sal, 30 DF

Comparisons of means for different levels of Date

Alpha 0.05 Standard Error for Comparison 0.2854

Critical T Value 2.117 Critical Value for Comparison 0.6042

Error terms used: Rep*Date and Rep*Date*Sal

The homogeneous group format can't be used

because of the pattern of significant differences.

**LSD All-Pairwise Comparisons Test of CndTotal for Rep**

**Rep Mean Homogeneous Groups**

2 0.1232 A

3 0.1157 A

4 0.1110 A

1 0.0989 A

Alpha 0.05 Standard Error for Comparison 0.0117

Critical T Value 2.179 Critical Value for Comparison 0.0255

There are no significant pairwise differences among the means.

**LSD All-Pairwise Comparisons Test of CndTotal for Date**

**Date Mean Homogeneous Groups**

1 0.1460 A

4 0.1393 A

3 0.0959 B

2 0.0943 B

5 0.0856 B

Alpha 0.05 Standard Error for Comparison 0.0131

Critical T Value 2.179 Critical Value for Comparison 0.0285

There are 2 groups (A and B) in which the means

are not significantly different from one another.

**LSD All-Pairwise Comparisons Test of CndTotal for Sal**

**Sal Mean Homogeneous Groups**

2 0.1180 A

1 0.1165 A

3 0.1021 B

Alpha 0.05 Standard Error for Comparison 6.942E-03

Critical T Value 2.042 Critical Value for Comparison 0.0142

There are 2 groups (A and B) in which the means

are not significantly different from one another.

**LSD All-Pairwise Comparisons Test of CndTotal for Date*Sal**

**Date Sal Mean Homogeneous Groups**

1 2 0.1664 A

4 1 0.1663 AB

4 2 0.1457 AB

1 3 0.1389 ABC

1 1 0.1327 BCD

4 3 0.1059 CDE

3 1 0.1056 CDE

2 1 0.1049 CDE

5 2 0.0986 DE

3 2 0.0944 DE

2 3 0.0932 E

3 3 0.0876 E

5 3 0.0851 E

2 2 0.0847 E

5 1 0.0731 E

Comparisons of means for the same level of Date

Alpha 0.05 Standard Error for Comparison 0.0155

Critical T Value 2.042 Critical Value for Comparison 0.0317

Error term used: Rep*Date*Sal, 30 DF

Comparisons of means for different levels of Date

Alpha 0.05 Standard Error for Comparison 0.0182

Critical T Value 2.113 Critical Value for Comparison 0.0385

Error terms used: Rep*Date and Rep*Date*Sal

There are 5 groups (A, B, etc.) in which the means

are not significantly different from one another.

Statistix 10.0 (30-day Trial) 9/11/2024, 2:18:20 PM

**Means of WUE for Rep**

**Rep Mean**

1 0.4962

2 0.4229

3 0.4436

4 0.4642

Observations per Mean 15

Standard Error of a Mean 0.0281

Std Error (Diff of 2 Means) 0.0397

Error term used: Rep*Date, 12 DF

**Means of WUE for Date**

**Date Mean**

1 0.3386

2 0.5026

3 0.6731

4 0.4488

5 0.3205

Observations per Mean 12

Standard Error of a Mean 0.0314

Std Error (Diff of 2 Means) 0.0444

Error term used: Rep*Date, 12 DF

**Means of WUE for Sal**

**Sal Mean**

1 0.4654

2 0.4385

3 0.4662

Observations per Mean 20

Standard Error of a Mean 0.0208

Std Error (Diff of 2 Means) 0.0294

Error term used: Rep*Date*Sal, 30 DF

**Means of WUE for Date*Sal**

**Date Sal Mean**

1 1 0.3709

1 2 0.3596

1 3 0.2852

2 1 0.4762

2 2 0.5139

2 3 0.5178

3 1 0.7105

3 2 0.6237

3 3 0.6850

4 1 0.3531

4 2 0.4226

4 3 0.5705

5 1 0.4161

5 2 0.2729

5 3 0.2725

Observations per Mean 4

Standard Error of a Mean 0.0465

Error term used: Rep*Date*Sal, 30 DF

**Means of Photo for Rep**

**Rep Mean**

1 8.7914

2 9.1819

3 9.4947

4 9.1184

Observations per Mean 15

Standard Error of a Mean 0.3983

Std Error (Diff of 2 Means) 0.5632

Error term used: Rep*Date, 12 DF

**Means of Photo for Date**

**Date Mean**

1 8.304

2 9.703

3 11.825

4 10.915

5 4.985

Observations per Mean 12

Standard Error of a Mean 0.4453

Std Error (Diff of 2 Means) 0.6297

Error term used: Rep*Date, 12 DF

**Means of Photo for Sal**

**Sal Mean**

1 9.7986

2 9.3095

3 8.3317

Observations per Mean 20

Standard Error of a Mean 0.3332

Std Error (Diff of 2 Means) 0.4711

Error term used: Rep*Date*Sal, 30 DF

**Means of Photo for Date*Sal**

**Date Sal Mean**

1 1 8.217

1 2 9.978

1 3 6.718

2 1 10.350

2 2 9.224

2 3 9.535

3 1 13.553

3 2 11.324

3 3 10.598

4 1 11.368

4 2 10.867

4 3 10.510

5 1 5.505

5 2 5.154

5 3 4.297

Observations per Mean 4

Standard Error of a Mean 0.7449

Error term used: Rep*Date*Sal, 30 DF

**Means of Trmmol for Rep**

**Rep Mean**

1 1.9344

2 2.2534

3 2.1709

4 2.0620

Observations per Mean 15

Standard Error of a Mean 0.1334

Std Error (Diff of 2 Means) 0.1886

Error term used: Rep*Date, 12 DF

**Means of Trmmol for Date**

**Date Mean**

1 2.4638

2 1.9758

3 1.8350

4 2.6149

5 1.6363

Observations per Mean 12

Standard Error of a Mean 0.1491

Std Error (Diff of 2 Means) 0.2109

Error term used: Rep*Date, 12 DF

**Means of Trmmol for Sal**

**Sal Mean**

1 2.2365

2 2.1773

3 1.9017

Observations per Mean 20

Standard Error of a Mean 0.0745

Std Error (Diff of 2 Means) 0.1054

Error term used: Rep*Date*Sal, 30 DF

**Means of Trmmol for Date*Sal**

**Date Sal Mean**

1 1 2.2700

1 2 2.7739

1 3 2.3474

2 1 2.2236

2 2 1.8014

2 3 1.9023

3 1 1.9909

3 2 1.8281

3 3 1.6860

4 1 3.2479

4 2 2.6172

4 3 1.9797

5 1 1.4500

5 2 1.8660

5 3 1.5930

Observations per Mean 4

Standard Error of a Mean 0.1666

Error term used: Rep*Date*Sal, 30 DF

**Means of CndTotal for Rep**

**Rep Mean**

1 0.0989

2 0.1232

3 0.1157

4 0.1110

Observations per Mean 15

Standard Error of a Mean 8.267E-03

Std Error (Diff of 2 Means) 0.0117

Error term used: Rep*Date, 12 DF

**Means of CndTotal for Date**

**Date Mean**

1 0.1460

2 0.0943

3 0.0959

4 0.1393

5 0.0856

Observations per Mean 12

Standard Error of a Mean 9.243E-03

Std Error (Diff of 2 Means) 0.0131

Error term used: Rep*Date, 12 DF

**Means of CndTotal for Sal**

**Sal Mean**

1 0.1165

2 0.1180

3 0.1021

Observations per Mean 20

Standard Error of a Mean 4.909E-03

Std Error (Diff of 2 Means) 6.942E-03

Error term used: Rep*Date*Sal, 30 DF

**Means of CndTotal for Date*Sal**

**Date Sal Mean**

1 1 0.1327

1 2 0.1664

1 3 0.1389

2 1 0.1049

2 2 0.0847

2 3 0.0932

3 1 0.1056

3 2 0.0944

3 3 0.0876

4 1 0.1663

4 2 0.1457

4 3 0.1059

5 1 0.0731

5 2 0.0986

5 3 0.0851

Observations per Mean 4

Standard Error of a Mean 0.0110

Error term used: Rep*Date*Sal, 30 DF
